# Genetic diversity of wild-type measles viruses: implications for global measles elimination programs.

**DOI:** 10.3201/eid0401.980105

**Published:** 1998

**Authors:** W J Bellini, P A Rota

**Affiliations:** Centers for Disease Control and Prevention, Atlanta, Georgia 30333, USA. wjb2@cdc.gov

## Abstract

Wild-type measles viruses have been divided into distinct genetic groups according to the nucleotide sequences of their hemagglutinin and nucleoprotein genes. Most genetic groups have worldwide distribution; however, at least two of the groups appear to have a more limited circulation. To monitor the transmission pathways of measles virus, we observed the geographic distribution of genetic groups, as well as changes in them in a particular region over time. We found evidence of interruption of indigenous transmission of measles in the United States after 1993 and identified the sources of imported virus associated with cases and outbreaks after 1993. The pattern of measles genetic groups provided a means to describe measles outbreaks and assess the extent of virus circulation in a given area. We expect that molecular epidemiologic studies will become a powerful tool for evaluating strategies to control, eliminate, and eventually eradicate measles.


### Genetic Diversity of Wild-Type Measles Viruses: Implications for Global Measles Elimination Programs

**William J. Bellini and Paul A. Rota**
Centers for Disease Control and Prevention, Atlanta, Georgia, USA

Wild-type measles viruses have been divided into distinct genetic groups according to the nucleotide sequences of their hemagglutinin and nucleoprotein genes. Most genetic groups have worldwide distribution; however, at least two of the groups appear to have a more limited circulation. To monitor the transmission pathways of measles virus, we observed the geographic distribution of genetic groups, as well as changes in them in a particular region over time. We found evidence of interruption of indigenous transmission of measles in the United States after 1993 and identified the sources of imported virus associated with cases and outbreaks after 1993. The pattern of measles genetic groups provided a means to describe measles outbreaks and assess the extent of virus circulation in a given area. We expect that molecular epidemiologic studies will become a powerful tool for evaluating strategies to control, eliminate, and eventually eradicate measles.

Until the advent of a live-attenuated vaccine in the early 1960s, measles was an epidemic disease worldwide. Today many countries have controlled measles, but the disease remains endemic on most continents. Development of a live-attenuated measles vaccine and implementation of laws that required proof of vaccination upon school entry dramatically reduced the incidence of measles in the United States. The number of reported cases plummeted from approximately 500,000 before vaccine introduction in 1963 to fewer than 1,500 in 1983. Despite these measures, a reemergence or resurgence of measles in the United States from 1989 to 1991 resulted in more than 55,000 cases of measles and approximately 120 measles-associated deaths (Figure 1; 1). In exploring the reasons for the resurgence, our laboratory genetically characterized measles viruses isolated from wild-type virus-infected persons from the same outbreak or temporally and geographically distinct outbreaks in the United States; in the regions examined, all measles viruses isolated during the period of resurgence were almost identical in nucleotide sequence and genetically distinct from vaccine strains (2).

Measles isolates from many regions of the world have been characterized in parallel studies by our laboratory and by others. In conjunction with classic epidemiologic investigations, these well-characterized viruses have formed a picture of the distribution of wild-type measles viruses in most areas of the world. Eight distinct genotypes have been identified, and undoubtedly more will be added. Some are localized to specific regions, while most are widely distributed. The assembly

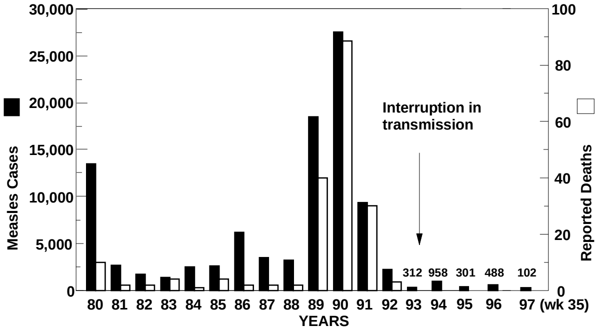

Figure 1. Incidence of U.S. measles cases and measles deaths between 1980 and 1997. Total number of measles cases for years 1993-1997 (week 35) are indicated above each bar.

Address for correspondence: William J. Bellini, MS C-22, Centers for Disease Control and Prevention, 1600 Clifton Rd., Atlanta, GA, 30333 USA; fax: 404-639-4187; e-mail: wjb2@cdc.gov.

of these sequences into a large database, which includes their geographic distribution, has become a new means by which measles transmission pathways can be traced and control measures can be assessed. This "molecular epidemiology" has affected the U.S. measles elimination program, and if used appropriately with standard epidemiologic methods, it will affect global measles elimination and eradication. This article summarizes the status of the known measles genotype distribution throughout the world and describes how molecular epidemiologic information has been used to assess the effectiveness of measles elimination in the United States.

### Global Distribution of Measles Genotypes

In most cases, genetic characterization of wild-type measles viruses has been conducted by sequencing the genes coding for the hemagglutinin (H) protein or the nucleoprotein (N). Of the six genes on the viral genome, the H and N genes are the most variable. Over their protein coding regions, the H and N genes vary by up to 7% at the nucleotide level. The single most variable part of the measles genome is the 450 nucleotides that code for the COOH-terminus of the N protein, where nucleotide variability between various wild-type viruses can approach 12%. Several laboratories have analyzed the sequences of wild-type measles viruses and assigned the viruses to various genetic groups (2-14). At present, no widely accepted standard is available for describing genetic groups of measles virus. However, with a few exceptions, the assignment of viruses to a particular group has been consistent between laboratories (10).

Many of our studies have focused on the genetic characterization of measles viruses associated with cases and outbreaks in the United States during the last 10 years (2,11). These viruses can be separated into at least eight distinct genetic groups (Table; Figure 2). Phylogenetic analyses using various computer programs (15-17) indicated good statistical support for each of the groups described below. Actually, more than eight genetic groups are listed when viruses from groups not yet found in the United States are included (e.g., Zambia: 1993, Germany: 1992) (Figure 2). The number of genetic groups is likely to increase since the true extent of genetic heterogeneity among wild-type measles viruses is still unknown, and virologic surveillance has not been conducted or has only just begun in many areas of the world.

Table. Sources of genotypes isolated in the United States, 1995–1997

| Group | No. of isolates | Imports from | Also circulating in |
|---|---|---|---|
| 4 | 13 | Germany, Spain, United Kingdom, Brazil, Austria, Italy, Greece, Ukraine | Central Europe, Canada, Brazil, United Kingdom |
| 3 | 9 | Japan | Japan, Thailand |
| 5 | 8 | Italy, Germany | Central Europe, United Kingdom, Brazil |
| 7 | 3 | Pakistan, Kenya | South Africa, Canada |
| 8 | 2 | China, Vietnam | China |
| 2 | 1 | Philippines | United States 1989–1992, Micronesia |
| 1 | 1 | Unknown | United Kingdom, Russia, China, Argentina |
| 6 | 1 | Kenya | The Gambia, Cameroon, Gabon, Zambia |

Group 1 contains the prototype, Edmonston strain, which was isolated in 1954. This group also contains all vaccine viruses sequenced regardless of whether they were derived from Edmonston (Attenuvax, Edmonston-Zagreb, AIKC, Schwarz) or from temporally and geographically independent wild-type isolates (Shanghai-191: China, Changchun-47: China, CAM-70: Japan, Leningrad-16: Russia) (18). Relatively few wild-type viruses from the prevaccine era are available for molecular characterization. These viruses, which were isolated in Japan, Russia, Finland, Romania, and the United States during the 1950s and 1960s, are in group 1 (9). Therefore, while viruses belonging to the other genetic groups may have been present, group 1 viruses must have had widespread distribution during the prevaccine era. Group 1 viruses continue to circulate, and viruses from group 1 were isolated from patients with clinical measles in the United States, United Kingdom, Russia, China, and Argentina during the last 7 years (5,11,19,20, and unpub. observations). These recent group 1 wild-type

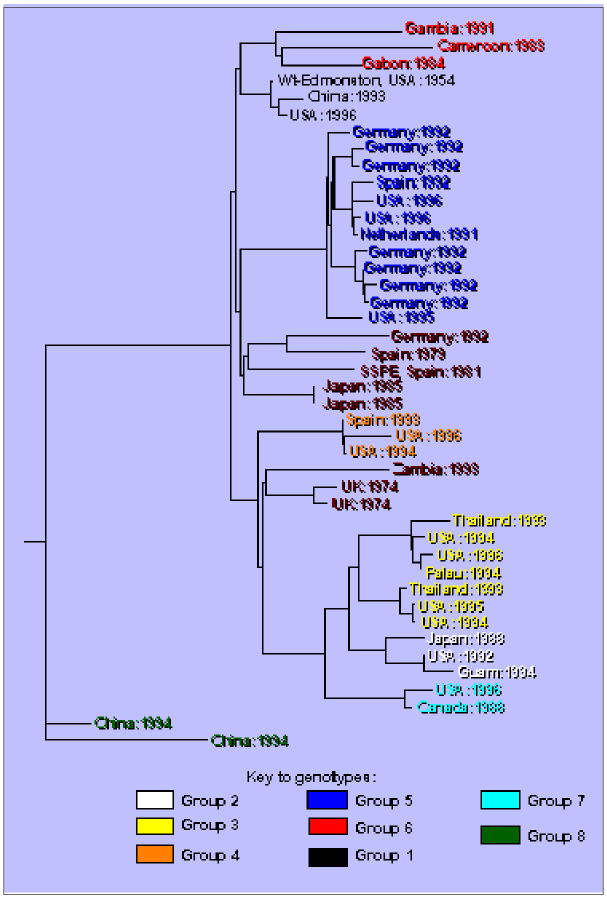

Figure 2. Phylogenetic tree showing genetic relationships between the eight genetic groups of measles virus associated with U.S. outbreaks and cases since 1988. The location and year of isolation is given for each virus. Viruses not assigned to one of the eight groups are labeled in brown. The unrooted tree is based on the sequence of the protein coding region of the H gene (1854 nt). Wt-Edmonston = low passage seed of the original Edmonston isolate. SSPE = sequences obtained from cases of subacute sclerosing panencephalitis.

viruses have several nucleotide substitutions that distinguish them from vaccine viruses. In contrast, measles vaccine viruses reisolated from immunosuppressed patients with giant cell pneumonia had nucleotide sequences nearly identical to those of the vaccine virus found in the vaccine vial (unpub. observations). This suggests that vaccine viruses are very stable even after prolonged replication in a human host. Therefore, it is unlikely that the group 1 wild-type viruses represent laboratory contamination of cultures with vaccine virus or reisolation of vaccine virus from recently vaccinated persons. Sequence studies have failed to identify a distinct set of genetic markers that consistently differentiate wild-type and presumably virulent viruses from attenuated viruses. Current studies are focusing on the analysis of the noncoding regions of the viral genome. More studies are needed to compare attenuated strains with their more virulent or reactogenic precursors. The recent development of an infectious clone for measles (21) will, no doubt, contribute to those studies.

Group 2 viruses were associated with the resurgence of measles in the United States between 1989 to 1991, an epidemic that had an unusually high incidence of deaths and hospitalizations (Figure 1). The circulation of group 2 viruses within the United States was interrupted in 1993, and this will be described in more detail below. Among these viruses, the Illinois-1 (Chicago-1) strain has become a representative of recent wild-type viruses, and almost the entire genome has been sequenced. Group 2 viruses were first isolated in Japan during the early 1980s; more recently, they were isolated in Japan, the Philippines, and Micronesia (2,11-13,22).

The group referred to as group 3 viruses can actually be divided into two distinct groups with a common geographic distribution. These viruses have been isolated from outbreaks in Japan and Thailand and from sporadic cases following importation into North America and Europe (2,11). Although virus from groups 2 and 3 cocirculated in Japan during the late 1980s and early 1990s, the group 3 viruses have recently become the predominant genotype (23).

Groups 4 and 5 appear to be circulating widely in many countries in western Europe, particularly Germany, Spain, and the United Kingdom, where virologic surveillance has been conducted (6,7,24). Viruses from this group are also circulating in France, Italy, Austria, and Greece since they have been associated with multiple importations from these areas into the United States (2,11; Table).

All representatives of the group 6 viruses have been isolated in the African countries of The Gambia (4), Cameroon, Gabon, and Zambia or associated with importations into the United States from Kenya. There is more genetic variability within the group 6 viruses than among most of the other genetic groups, yet all group 6 viruses contain a subset of nucleotide substitutions that places them on this African lineage.

Relatively few viruses from central Africa, where most measles infections are occurring, have been isolated for genetic analysis, and it will be interesting to determine if other genotypes are also present in this area.

Group 7 viruses were first isolated during an epidemic in Montreal, Canada, in 1988 (2,11). Group 7 was the predominant group among a number of recently isolated viruses from Johannesburg, South Africa (9,25,26). The identification of a group 7 virus in association with an importation to the United States from Pakistan suggests (11) that the viruses in this group may be circulating widely in Africa and Asia.

The group 8 viruses form a highly distinct group isolated in four provinces within the People's Republic of China during the early 1990s (19). Like group 6, group 8 viruses have more nucleotide variability within the group (up to 3%) than the other groups. Recent evidence also suggests that group 8 viruses are circulating in other parts of China (Hong Kong) and in Vietnam.

Several recently isolated viruses do not fit into the eight genetic groups that, so far, contain most recent isolates (Figure 2). Some outliers represent single isolations of a unique genetic type. However, preliminary analysis of a number of wild-type viruses from Zambia indicates that these viruses belong to a genetic group that is distinct from the eight groups described thus far (unpub. observations).

If viruses isolated during the early to mid-1980s were included into the genetic analysis (not shown), it would be apparent that more genetic groups exist. However, viruses representing these groups have not been isolated in the last 10 to 15 years, and it must be assumed that these groups are circulating in restricted geographic regions, are circulating at such a low frequency as to escape surveillance, or are extinct.

A summary of genetic groups (Figure 3) represents a static picture that simply identifies where particular genetic groups have been isolated, with no accounting for frequency of isolation or source of the virus. Certain regions of the world (including much of Africa and most of southern Asia) are still vastly underrepresented. A survey of recent Australian isolates is in progress. The pattern of genotypes in the United Kingdom and the United States is very complex because of relatively good strain surveillance and the frequency of international travel to these locations.

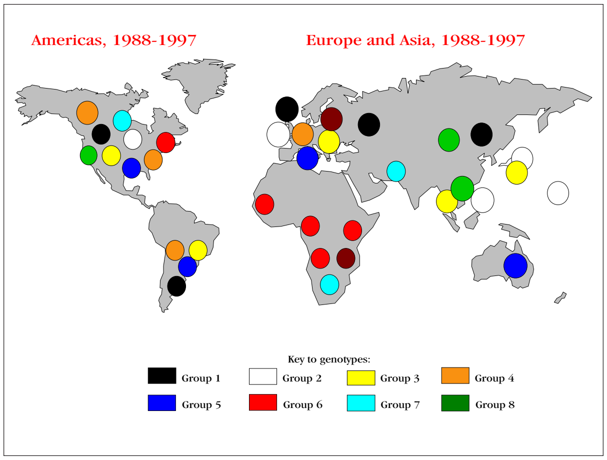

Figure 3. Global distribution of measles genetic groups. Colored circles indicate areas where measles viruses from various genetic groups have been isolated. Viruses not assigned to one of the eight groups are labeled in brown.

### Molecular Epidemiology

Measles has long been considered one of the most communicable of diseases. The resurgence of disease from 1989 to 1991 in the United States (Figure 1) provides a good example of the rapid transmissibility of the virus. During this resurgence only group 2 viruses were isolated, and the sequences from these viruses were highly related (2). With continued molecular surveillance, we were able to document the interruption of transmission of the group 2 viruses and monitor the change in measles genotypes associated with outbreaks and sporadic cases from 1994 to the present. Molecular surveillance of measles viruses was most useful when the change in genotypes was observed over time. Without that information, it would not have been possible to describe the transition from an apparently "indigenous" lineage to importation of multiple lineages (Figure 4). This is in contrast to the situation in South and Central America. In these areas, viral surveillance was not conducted before mass vaccination campaigns were initiated, so the identity of the prevailing genotype could not be determined. Therefore, it is difficult to interpret the genetic data obtained from viruses currently causing outbreaks in these regions.

The molecular surveillance of wild-type viruses in the United States between 1989 and

1997 provides the best example of dynamic molecular surveillance (Figure 4). Viruses isolated over a 4-year period from major outbreaks in New York, Philadelphia, Chicago, Los Angeles, Houston, and southern Texas varied by less than 0.4% at the nucleotide level in the H and N genes (2). Analysis of the few wild-type measles viruses isolated in the United States before 1988 indicates lineages other than group 2. This suggests that the group 2 viruses were probably imported during the late 1980s and were rapidly transmitted to the entire country. While numerous importations occurred during the resurgence, apparently these viruses did not circulate widely enough to be detected by molecular surveillance. Perhaps the number of measles-susceptible persons in the U.S. population during the resurgence was high enough to sustain continuous transmission without accumulation of variants or displacement by other imported viruses.

More aggressive childhood vaccination programs, the introduction of a two-dose schedule, and successful mass vaccination campaigns conducted by the Pan American Health Organization in South and Central America greatly reduced the number of reported measles cases in the United States in 1993 (Figure 1). During a 6-week period at the end of 1993, no indigenous cases of measles were reported (27). Molecular surveillance of measles viruses associated with cases and outbreaks in the United States during 1994, 1995, 1996, and 1997 documented this interruption of transmission of what had been the indigenous genotype (2,11). Only one group 2 virus was detected in the United States after 1993, and this was directly linked to importation from the Philippines (11).

Molecular surveillance data allow us to draw several conclusions about the transmission of measles virus in the United States. The first is that increasing the level of population immunity by vaccination can interrupt the transmission of measles virus. This is hardly new information, and interruption of transmission was described for The Gambia in 1983 and more recently in

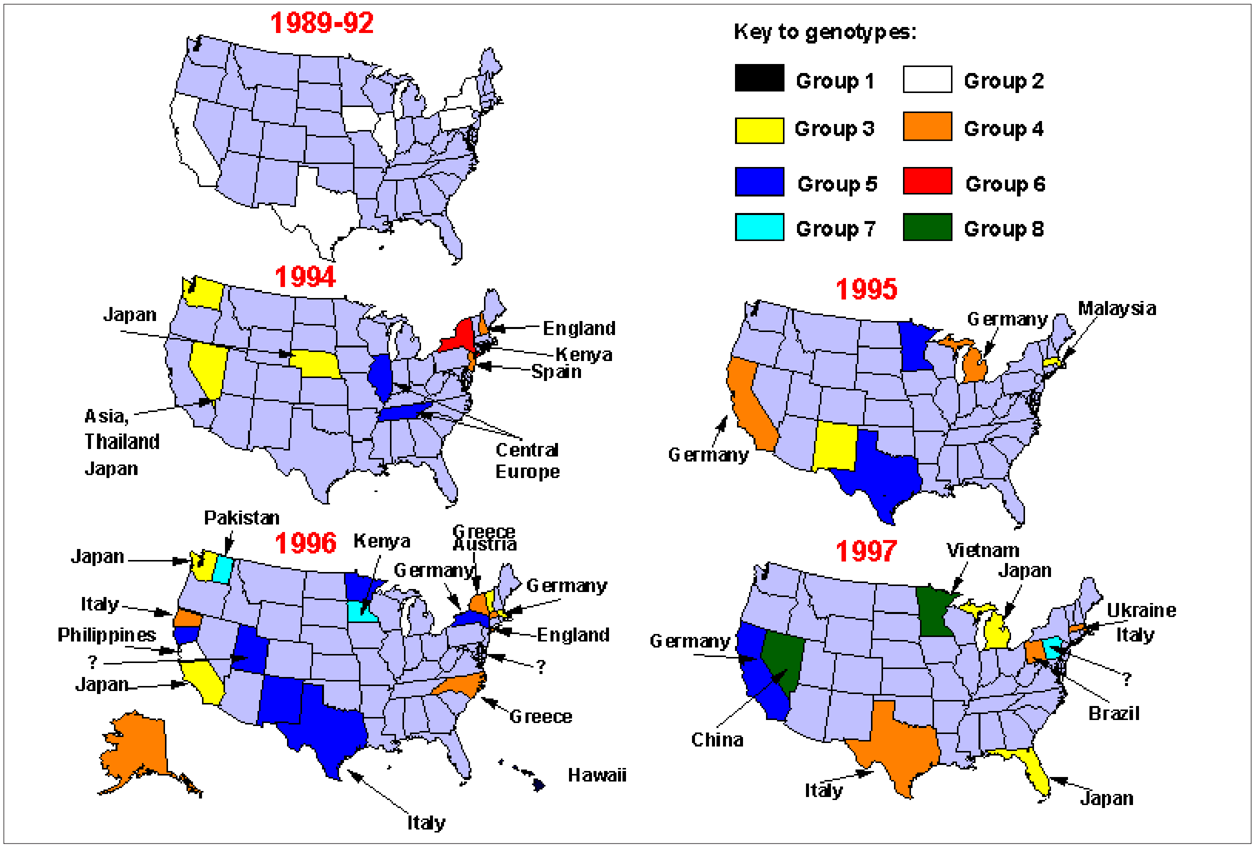

Figure 4. Change in genetic groups of measles viruses associated with U.S. cases and outbreaks between 1988 and September 1997. Arrows indicate sources of virus, if known.

Finland. However, our studies are the first in which genetic analysis of measles strains has been used to document interruption of transmission. Secondly, long-term asymptomatic transmission of virus is unlikely since no group 2 viruses were detected in the United States after 1993 that were not directly linked to importation. Finally, measles will not be fully controlled anywhere until it is controlled globally. Virus introduced by importation will continue to fuel sporadic outbreaks and epidemics even in areas with relatively good control measures. These observations should strengthen our resolve to accelerate measles control activities on a global level.

The molecular data imply that under conditions of continuous indigenous transmission of measles virus, the number of circulating genotypes is limited. As population immunity increases, the pattern of genotypes becomes more complex to reflect the multiple sources of imported virus. We hope to test this model further by conducting molecular surveillance of wild-type measles viruses circulating in areas that still have endemic measles.

### Conclusions

Genetic characterization of wild-type measles viruses provides a valuable means to measure the level of virus circulation in areas just beginning to implement measles control plans. In areas that already achieved good measles control, molecular epidemiologic studies provide a means to describe outbreaks and cases. Identifying the source of the virus can lead to improved control measures. To be maximally effective, molecular epidemiologic studies must include surveys of viral genetic groups from all areas of the world. Specimens for viral isolations should be obtained from as many chains of transmission as possible. Obtaining specimens must become an integral part of measles surveillance and be included in the standard operating procedures for investigating measles cases. If we can establish a large database to describe the indigenous genetic groups before large-scale control measures are enacted, we can closely monitor the ability of these control measures to reduce or interrupt transmission of measles. Molecular epidemiology will greatly enhance measles elimination and eradication efforts.

Dr. Bellini is chief of the Measles Section, Respiratory and Enteric Viruses Branch, Division of Viral and Rickettsial Diseases, National Center for Infectious Diseases, CDC. Dr. Rota is a microbiologist in the Measles Section, CDC. The authors work closely with national and international public health agencies to support global measles eradication activities. They provide expertise and training on the genetic characterization of measles viruses and laboratory diagnosis of measles infections. Research interests include molecular virology, viral pathogenesis, and immune response to viral infections.

### References

1. Atkinson WL, Orenstein WA, Krugman S. The resurgence of measles in the United States, 1989-1990. Annu Rev Med 1992;43:451-63
2. Rota JS, Heath JL, Rota PA, King GE, Celma ML, Carabaña J, et al. Molecular epidemiology of measles virus: identification of pathways of transmission and implications for measles elimination. J Infect Dis 1996;173:32-7.
3. Kreiss S, Whistler T. Rapid identification of measles virus strains by the heteroduplex mobility assay. Virus Res 1997;47:197-203.
4. Outlaw MC, Jaye A, Whittle H, Pringle C. Clustering of hemagglutinin sequences of measles viruses isolated in the Gambia. Virus Res 197;48:125-31.
5. Outlaw MC, Pringle C. Sequence variation within an outbreak of measles virus in the Coventry area during spring/summer 1993. Virus Res 1995;39:3-11.
6. Rima BK, Earle JAP, Yeo RP, Herlihy L, Baczko K, ter Meulen V, et al. Temporal and geographical distribution of measles virus genotypes. J Gen Virol 1995;76:1173-80.
7. Rima BK, Earle JAP, Baczko K, ter Meulen V, Liebert U, Carstens C, et al. Sequence divergence of measles virus haemagglutinin during natural evolution and adaptation to cell culture. J Gen Virol 1997;78:97-106.
8. Rima B, Earle JAP, Baczko K, Rota PA, Bellini WJ. Measles virus strain variations. Current Topics Microbiol Immunol 1995;191:65-84.
9. Rota PA, Bloom AE, Vanchiere JA, Bellini WJ. Evolution of the nucleoprotein and matrix genes of wild-type strains of measles virus isolated from recent epidemics. Virology 1994;198:724-30.
10. Rota PA, Rota JS, Bellini WJ. Molecular epidemiology of measles virus. Seminars in Virology 1995;6:379-86.
11. Rota JS, Rota PA, Redd SC, Pattamadilok S, Bellini WJ. Phylogenetic analysis of measles viruses isolated in the United States 1995-1996. J Infect Dis. In press 1997.
12. Saito H, Sato H, Abe M, Harata S, Amano K, Suto T, et al. Cloning and characterization of the cDNA encoding the HA protein of a hemagglutination-defective measles virus strain. Virus Genes 1994;8;2:107-13.
13. Sakata H, Kobune F, Sato TA, Tanabayashi K, Yamada A, Sugiura A. Variation in field isolates of measles virus during an 8-year period in Japan. Microbiol Immunol 1993;37:233-7.

14. Taylor MJ, Godfrey E, Baczko K, ter Meulen V, Wild TF, Rima BK. Identification of several different lineages of measles virus. J Gen Virol 1991;72:83-8
15. Devereaux J, Haeberli P, Smithies O. A comprehensive set of sequence analysis programs for the VAX. Nucleic Acids Res 1984;12:387-95.
16. Felsenstein J. Phylogenies from molecular sequences; inferences and reliability. American Review of Genetics 1988;22:512-65.
17. Swofford DL. PAUP: phylogenetic analysis using parsimony [computer program]. Version 3.1.1. Champaign (IL): Illinois Natural History Survey; 1991.
18. Rota JS, Wang ZD, Rota PA, Bellini WJ. Comparison of sequences of the H, F, and N coding genes of measles virus vaccine strains. Virus Res 1994;31:317-30.
19. Xu WB, Tamin A, Rota JS, LiBi J, Bellini WJ, Rota PA. New genetic group of measles virus isolated in the People's Republic of China. Virus Res. In press 1998.
20. Heider A, Santibanez S, Tischer A, Gerike E, Tikhonova N, Ignatyev G, et al. Comparative investigation of the long non-coding M-F genome region of wild-type and vaccine measles viruses. Arch Virol. In press 1998.
21. Radecke F, Speilhofer P, Schneider H, Kaelin K, Huber M, Dotsch C, et al. Rescue of measles virus from cloned DNA. EMBO J 1995;14:5773-84.
22. Guris D, Auerbach S, Vitek C, Maes E, McCready J, Durand M, et al. Measles outbreaks in Micronesia, 1991-1994. J Pediatr. In press 1997.
23. Katayama Y, Shibahara K, Kohama T, Homma M, Hotta H. Molecular epidemiology and changing distribution of genotypes of measles virus field strains in Japan. J Clin Microbiol 1997;35:2651-3.
24. Jin L, Brown DWG, Ramsay MEB, Rota PA, Bellini WJ. The diversity of measles virus in the UK, 1992-1995. J Gen Virol 1997;78:1287-94.
25. Kreis S, Whistler T. Rapid identification of measles virus strains by the heteroduplex mobility assay. Virus Res 1997;47:197-203.
26. Kreis S, Vardas E, Whistler T. Sequence analysis of the nucleocapsid gene of measles virus isolates from South Africa identifies a new genotype. J Gen Virol 1997;78:1581-7.
27. Centers for Disease Control and Prevention. Absence of reported measles—United States, November 1993. MMWR Morb Mortal Wkly Rep 1993;42(48):925-6.
